# Ceramide and Its Related Neurochemical Networks as Targets for Some Brain Disorder Therapies

**DOI:** 10.1007/s12640-017-9798-6

**Published:** 2017-08-25

**Authors:** Justyna Brodowicz, Edmund Przegaliński, Christian P. Müller, Malgorzata Filip

**Affiliations:** 1 Faculty of Pharmacy, Jagiellonian University Medical College, 9 Medyczna Street, 30-688 Kraków, Poland; 2 Department of Drug Addiction Pharmacology, Institute of Pharmacology, Polish Academy of Sciences, 12 Smętna Street, 31-343 Kraków, Poland; 3 Department of Psychiatry and Psychotherapy, University Clinic, Friedrich-Alexander-University of Erlangen-Nuremberg, Schwabachanlage 6, 91054 Erlangen, Germany

**Keywords:** Ceramide, Sphingolipids, Psychiatric disorders, Neurological disorders, Clinical trials, Preclinical research

## Abstract

Correlational and causal comparative research link ceramide (Cer), the precursor of complex sphingolipids, to some psychiatric (e.g., depression, schizophrenia (SZ), alcohol use disorder, and morphine antinociceptive tolerance) and neurological (e.g., Alzheimer’s disease (AD), Parkinson disease (PD)) disorders. Cer generation can occur through the de novo synthesis pathway, the sphingomyelinase pathways, and the salvage pathway. The discoveries that plasma Cer concentration increase during depressive episodes in patients and that tricyclic and tetracyclic antidepressants functionally inhibit acid sphingomyelinase (ASM), the enzyme that catalyzes the degradation of sphingomyelin to Cer, have initiated a series of studies on the role of the ASM-Cer system in depressive disorder. Disturbances in the metabolism of Cer or SM are associated with the occurrence of SZ and PD. In both PD and SZ patients, the elevated levels of Cer or SM in the brain regions were associated with the disease. AD patients showed also an abnormal metabolism of brain Cer at early stages of the disease which may suggest Cer as an AD biomarker. In plasma of AD patients and in AD transgenic mice, ASM activity was increased. In contrast, partial ASM inhibition of Aβ deposition improved memory deficits. Furthermore, in clinical and preclinical research, ethanol enhanced activation of ASM followed by Cer production. Limited data have shown that Cer plays an important role in the development of morphine antinociceptive tolerance. In summary, clinical and preclinical findings provide evidence that targeting the Cer system should be considered as an innovative translational strategy for some brain disorders.

## Sphingolipids—Metabolism and Functions

**Fig. 1:**
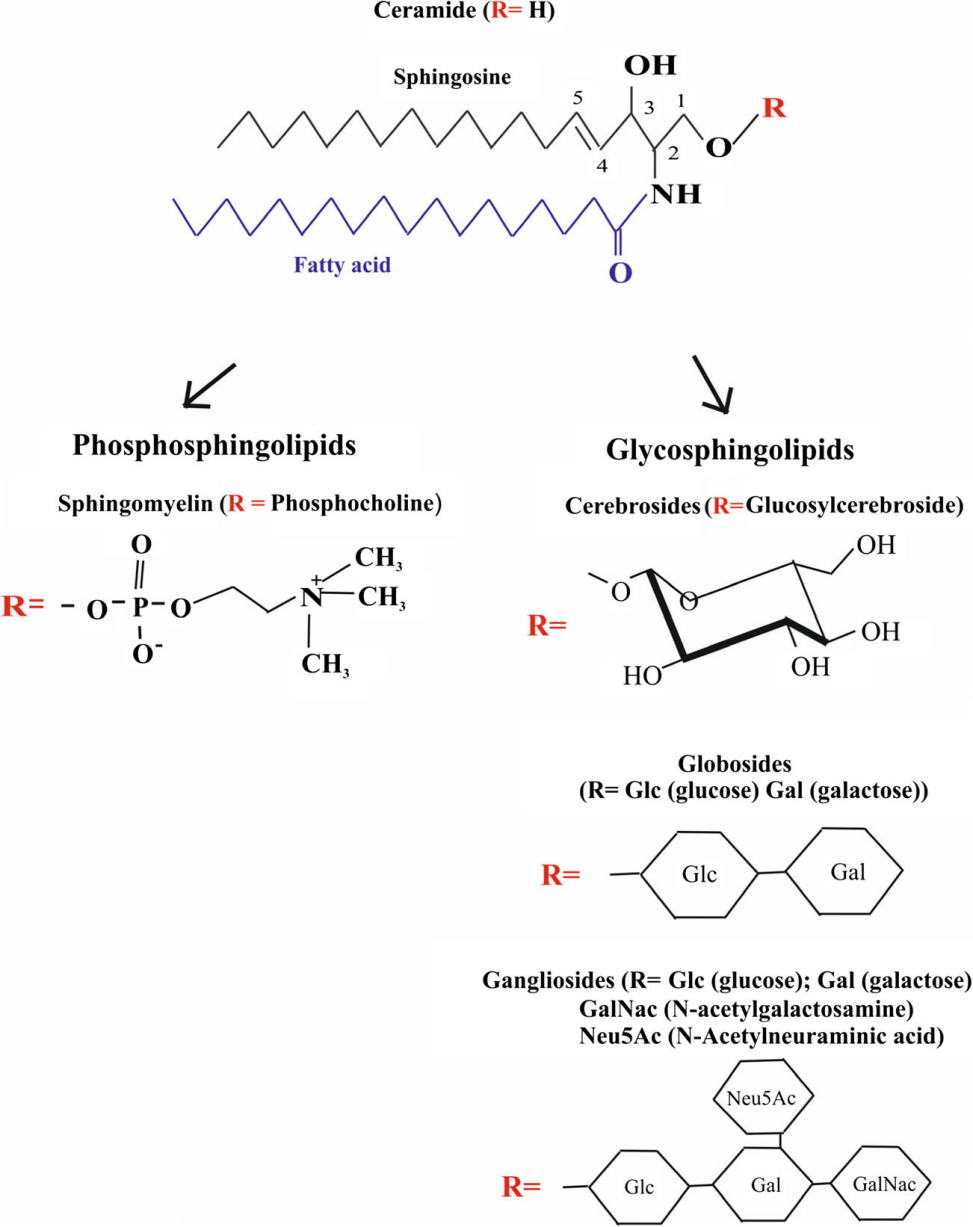
Chemical structure and representatives of the two main classes of sphingolipids [1, 6]

Sphingolipids (SLs) are a large group of amphipathic lipids which are found in abundance in cellular membranes. The name “SLs” was introduced by Herbert Carter and co-workers in 1947, followed by the root term “sphingo-“coined by J.L.W. Thudichum in 1884 to mark the “sphinx” (mystical)-like nature of the molecules. SLs have a hydrophobic nonpolar tail consisting of a fatty acid, a sphingoid base (e.g., sphinganine, sphingosine, or phytosphingosine) and a polar hydrophilic head. SLs are a very diverse group of lipids and can be classified according to the structural combination of long-chain sphingoid bases, by the length of the fatty acids, and by the various polar head groups. Depending on the type of polar head group, there are two major classes of SLs: phosphosphingolipids (PSLs), including sphingomyelin (SM), and glycosphingolipids (GSLs), including cerebrosides, gangliosides, and globosides (Fig. 1) (Mencarelli and Martinez-Martinez 2013).

SLs play an important structural role in biological membranes and yield many bioactive metabolites that regulate cell function. In the central nervous system, SLs are located in the cell membranes of nerve cells. In particular, SM is abundantly found in the myelin sheaths of nerve fibers. Gangliosides and globosides are mainly located in glial cells, but also present in neurons, while cerebrosides are present in glial cells (Yamaji and Hanada 2015). Of note, SM appears as most abundant SL in the brain. SM is also the major reservoir for ceramide formation in the cell (Gault et al. 2010).

## Ceramide

The most common alcohol in all SLs is unsaturated 18 carbon amino alcohol 1,3-dihydroxy-2-amino-4-octadecene called sphingosine. The combination of a fatty acid being a saturated or unsaturated fatty acid with up to 24 carbon atoms with sphingosine constitutes one of the functionally most important molecule species among the SLs—the ceramide (N-acylsphingosine, Cer) (Fig. 1) (Car et al. 2012; Mencarelli and Martinez-Martinez 2013). Cer is present in (i) SM, formed by the transfer of the phosphorylcholine moiety to a hydroxyl group on the first carbon atom of Cer; (ii) cerebrosides formed by the linking of the β-glycosidic bond of one or more sugar (glucose or galactose) residues to the hydroxyl group at the first Cer carbon; (iii) globosides being the cerebrosides which contain additional saccharide mainly galactose, glucose, or N-acetylgalactosamine; and (iv) gangliosides consisted of the Cer backbone esterified with three or more sugar residues of which one is sialic acid (Fig. 1) (Garrett and Grisham 2012).

### Cer Synthesis Pathways

The synthesis of Cer can occur in three different ways. The first one is the de novo synthesis of Cer which takes place in the endoplasmic reticulum. It starts with the condensation of serine and palmitoyl-Co to 3*-*ketosphinganine via action of serine palmitoyltransferase. 3-Ketosphinganine is then reduced by the enzyme 3-ketosphinganine reductase to sphinganine, followed by acylation to dihydroceramide by Cer synthases (CerS) (Lahiri and Futerman 2007). In humans, six different Cer synthases (CerS1–6) have been found, which differ in their affinity for distinct acyl groups and in tissue distribution. Thus, CerS1 synthesizes C18-Cer and is mainly expressed in the brain neurons and at low density in skeleton muscles and tests (Mizutani et al. 2005). CerS1 knockout mice show decreased levels of gangliosides in cerebellum and forebrain, a foliation defect, progressive shrinkage, neuronal apoptosis in the cerebellum, and functional deficits like impaired exploration of novel objects, locomotion, and motor coordination (Ginkel et al. 2012). CerS2 is expressed in almost all tissues and is responsible for the synthesis of Cer formed from fatty acids having 20–26 carbon atoms in length (C20–C26 Cer) (Kihara 2016). In the brain, CerS2 is highly expressed in the myelin-formed oligodendrocytes, and disruption of *CerS2* in mice produces myelin disorders such as myelin sheath defects and cerebellar degeneration (Vonsy et al. 2009). CerS3 synthetizes C24-C26-Cer that is important for skin barrier formation and spermatogenesis. Disruption of CerS3 in mice produces skin barrier defects and neonatal lethality (Jennemann et al. 2012). CerS4 is expressed mainly in the skin, leukocytes, heart, liver, and lung while at lower density in the brain (Levy and Futerman 2010). It synthetizes C18–22 Cer containing fatty acids (Riebeling et al. 2003). CerS4 knockout mice display hair loss and epidermal tissue destruction (Ebel et al. 2014). CerS5 synthetizes C16-Cer and is highly expressed in white adipose tissue, testis, and lung while low expression was found in the gray and white matter of the brain. In mice, deficiency in CerS5 leads to resistance to high-fat diet-induced obesity with improved glucose tolerance and insulin sensitivity (Levy and Futerman 2010). Similarly to CerS5, the CerS6 product is C16-Cer. CerS6 is expressed in the intestine and the kidney, in the brain within the hippocampus, cortex, and in Purkinje cells (Ebel et al. 2013). CerS6 knockout mice exhibit defective hind limb grasping, habituation in the open field test, and exacerbation of experimental autoimmune encephalomyelitis. Following action of CerS and dihydroceramid formation, the last step of the de novo Cer pathway is the formation of a double bond in dihydroceramid by the action of the enzyme dihydroceramide desaturase (Kihara 2016).

The second Cer synthesis pathway refers to sphingolipid recycling, called the “salvage” pathway. In lysosomes or in late endosomes, complex SLs can be metabolized into sphingosine, which can be used (possibly in endoplasmic reticulum or in endoplasmic reticulum-associated membranes) through re-acylation to produce Cer (or its derivatives) under sphingomyelinases (SMase), cerebrosidases, ceramidases, and ceramide synthases actions (Kitatani et al. 2008).

The third Cer synthesis way is called the sphingomyelinase (SMase) pathway. In this pathway, Cer synthesis is generated from hydrolysis of sphingomyelin through the action of SMases (Jenkins et al. 2009). Subcellular location of this process is dependent on cell type and is mediated by different SMases in a highly pH-dependent way in the cell membrane or in endo-lysosomes. Depending on the pH, there are three main forms of SMase: neutral sphingomyelinase (NSM), acid sphingomyelinase (ASM), and alkaline sphingomyelinase (alkSM) (Goi and Alonso 2002). The Mg^2+^-dependent NSM is located in the plasma membrane, whereas Mg^2+^-independent NSM occurs in the cytosol. Several studies indicate that Mg^2+^-dependent NSM is a major source of the stress-induced production of Cer (Young et al. 2012). ASM is a lysosomal glycoprotein, catalyzing the degradation of sphingomyelin to Cer and phosphorylcholine. ASM has a pH optimum around 5 and is located in the acidic lysosomes. Depending on the location in the cell and binding of cations, there are two types of ASM: the Zn^2+^-independent lysosomal (L-ASM) and Zn^2+^-dependent secretory (S-ASM) (Kornhuber et al. 2015). A deficiency of ASM leads to the development of recessively inherited lysosomal storage disorder, Niemann-Pick disease types A and B (Schuchman 2010).

### Cer Metabolism Pathways

Cer is used—among others—for the production of SM. In fact, de novo synthesized Cer is transported by the Cer transfer protein (CERT) to the Golgi apparatus where it is involved in the synthesis of SM requiring sphingomyelin synthase. Cer can also be transferred through a vesicular transport to the Golgi apparatus, where it can be transformed by glucosy-lceramide synthase (GCS) to glucosylceramide (GlcCer) which belongs to the GSLs (Jenkins et al. 2009).

The phosphorylation of the free hydroxyl group on the first carbon chain of the sphingosine backbone of Cer by ceramide kinase (CerK) leads to the formation ceramid-1-phosphate (C1P) (Shinghal et al. 1993; Gómez-Muñoz 2004). Subcellular localization CerK is not fully understood; however, it was suggested that it may occur in the plasma membrane, Golgi apparatus, and cytoplasm (Mitsutake et al. 2004).

The main route of Cer degradation in a cell is the metabolism by ceramidases. There are five different enzymes which catalyze this reaction with distinct pH optimum: three forms of alkaline ceramidase, a neutral ceramidase, and an acid ceramidase. Isoforms of the enzyme show different affinity to fatty acids of various chain lengths and place of occurrence in the cell. Alkaline ceramidases occur in the ER/Golgi complex. The neutral isoform is present in the outer plasma membrane, and the acidic form is located in lysosomes (Lahiri and Futerman 2007). Following ceramidase actions, enhanced by interleukine-1β (IL-1β), nitric oxide (NO), low density lipoprotein (LDL), and platelet-derived growth factor (PDGF) (Zheng et al. 2006), sphingosine is created. Subsequently, it can be phosphorylated to sphingosine-1-phosphate (S1P) by sphingosine kinases (SK1 and SK2) (van Echten-Deckert and Herget 2006).

### Cer Functions

Cer is an important bioactive molecule that has been implicated in mediating or regulating many cellular processes (Angelica and Fong 2008). For example, Cer has emerged as an important effector in development and stress responses, regulation of autophagy, mediating the crosstalk with apoptosis. A rise in Cer formation in many types of cells, including nerve cells, results in toxicity expressed as pro-apoptotic actions. The consequence of this process is tissue injury. On the other hand, Cer low levels have trophic effects and promote survival after cell division. Thus, Cer exerts a positive effect on early growth and differentiation of cells. Cer in the plasma membrane has a structural role as it creates lipid rafts and microdomains in the lipid bilayer that cluster of receptors and other signaling molecules (Schenck et al. 2007).

Recent evidence suggests that coping with stress may be accompanied by an increase in Cer levels (Oliveira et al. 2016), while decline of Cer levels, due to fast acting adaptations in ASM and NSM activity, may be important for re-learning during the extinction of a previously rewarded operant behavior (Huston et al. 2016; Sonnino and Prinetti 2016).

It should be added that Cer can act as a signaling molecule in its own right, or it can be further processed to generate sphingosine and S1P. C1P and S1P also govern cell growth and proliferation, differentiation, migration, aging, apoptosis, and inflammation (Lahiri and Futerman 2007). Furthermore, C1P has an important role as either a signaling molecule (Gómez-Muñoz 2004) or an inhibitor of ASM to block apoptosis (Gómez-Muñoz et al. 2004). Interestingly, S1P, Cer, and SM can be linked to therapeutic effects in various brain disorders (see below).

### Mechanisms Activating the Cer System

Intracellular Cer levels can be enhanced by different stimuli that activate either the de novo synthesis pathway (oxidized LDL, cannabinoids) or the SMase pathway (cytokine activation, UV radiation, ionizing radiation) or both pathways (chemotherapeutic agents, tumor necrosis factor-α (TNF-α), Fas ligand, phorbol ester, melatonin, heat stress, oxidative stress (OS) (for review, see Angelica and Fong 2008).

One of the most important activator of Cer generation is OS, being induced by reactive oxygen species (ROS) and reactive nitrogen species (RNS). Exogenous sources of ROS, e.g., hydrogen peroxide (H_2_O_2_), superoxide (O_2_^·−^), and RNS, e.g., peroxynitrate (ONOO^−^), are environmental factors such as UV radiation, ionizing radiation, heat exposure, and xenobiotics (environmental pollutants and solvents). ROS and RNS are also generated by endogenous sources such as mitochondrial dysfunction, pro-inflammatory cytokines (e.g., IL-1, IL-6, and TNF-α), activated microglia, amyloid β (Aβ), Cer itself, cytochrome P-450, and by neurotransmitters. ROS formation in the brain is associated with increased Ca^2+^ concentration inside the nerve cells that may disrupt mitochondrial function, e.g., by enhancing mitochondrial permeability and release of ROS. Ca^2+^ can activate a number of enzymes, such as, e.g., nitic oxide synthase (NOS), phospholipase A_2_, and calpain, which may themselves induce ROS. Another source of ROS comes from the dopamine (DA) system as the DA metabolism includes a non-enzymatic pathway leading to the formation of H_2_O_2_ and O_2_^·−^ as well as enzymatic pathways, which generate H_2_O_2_ (Niedzielska et al. 2014). Also, viruses or bacteria infections can lead to a Cer release via the ASM stimulation, which by itself causes the activation of NADPH oxidase and ROS production (Li et al. 2012).

### Pharmacology of the Cer System

Several agents are known that alter the amount of Cer, which can be used for therapeutic purposes. Myriocin (2-amino-3,4-dihydroxy-2-(hydroxymethyl)-14-oxoicos-6-enoic acid) and dimethylsphingosine are potent inhibitors of serine palmitoyltransferase and of sphingosine kinases, respectively, and can be used to prevent de novo synthesis of Cer. Fumonisin B1 (2*S*,2′*S*)-2,2′-{[(5*S*,6*R*,7*R*,9*R*,11*S*,16*R*,18*S*,19*S*)-19-amino-11,16,18-trihydroxy-5,9-dimethylicosane-6,7-diyl]bis[oxy(2-oxoethane-2,1-diyl)]}disuccinic acid) has a structure similar to sphingosine and inhibits ceramide synthase. It blocks the production of Cer in the de novo synthesis and salvage pathway resulting in a reduction of Cer levels, but also in a rise of the concentration of sphingosine and S1P (Merrill et al. 1997). 3,3′-(1,4-Phenylene)*bis*[N-[4-(4,5-dihydro-1H-imidazol-2-yl)phenyl]-dihydrochloride-2-propenamide (GW4869) is a cell-permeable, potent, specific, non-competitive inhibitor of NSM (IC_50_ = 1 μM, rat brain), which does not affect ASM (Luberto et al. 2002). The other non-selective NSM inhibitors are 3-O-methyl-sphingomyelin (3-OMe-SM) and N-acetylcysteine [from (Lee et al. 2004)]. D-threo-1-phenyl-2-decanoylamino-3-morpholino-1-propanol (D-PDMP) is a glucosylceramide synthase inhibitor that prevents glycosylation of Cer (Hisaki et al. 2004).

There are few agents that function as physiological (or direct) inhibitors of the ASM. They include α-mangostin, L-α-phosphatidyl-D-myoinositol-3,5-biphosphate, AD2765, phosphatidyl-myo-inositol-3,4,5-triphosphate, SMA-7, and zoledronic acid (Kornhuber et al. 2010; Müller et al. 2015).

Apart from the direct ASM inhibitors, there are several drugs that act as functional inhibitors of ASM activity (FIASMA). Many studies have shown that some drugs may lead to decrease of enzyme activity by a partial degradation of the ASM enzyme (Kölzer et al. 2004; Gulbins et al. 2013; Kornhuber et al. 2011, 2014; Beckmann et al. 2014). All so far identified FIASMAs have at least one basic nitrogen atom, a molecular weight of not more than 500 kDa, weak alkalinity, and high lipophilicity. Recent structure-properties-activity relationship (SPAR) model showed that to cause functional inhibition of ASM the high value of the negative logarithm of the acid dissociation constant (pKa), and logP is necessary but not sufficient to inhibit ASM. Among FIASMAs, there are many tricyclic and tetracyclic antidepressants (amitriptyline, desipramine, doxepine, fluoxetine, imipramine, maprotilin, nortriptyline, paroxetine, and sertraline) (Kornhuber et al. 2011, 2014). The above discoveries have initiated a series of studies on the role of ASM in a major depression.

### The Cer System in Pathology and Therapy of Some Brain Disorders

It is believed that the pathology of some brain disorders such as depression, schizophrenia, substance use disorder, and pharmacotherapy of morphine antinociceptive tolerance as well as neurodegenerative disease (Alzheimer’s disease, Parkinson disease) can be explained by altered storage of Cer or SLs.

#### Depression

Depression is a psychiatric disorder characterized by key symptoms that include depressed mood and a loss of interest and pleasure. A major problem associated with depressive mood disorders is suicide with a death rate of 850,000 people per year (Lang and Borgwardt 2013). There are several hypotheses regarding the etiology of depression, which include a deficiency in the noradrenergic, serotonergic, and/or DA-ergic systems. Other hypotheses of depression concern the role of chronic stress, neurotrophic factors, inflammatory cytokine, GABA-ergic, and glutamatergic network systems (Palazidou 2012; Caddy et al. 2014).

Some clinical studies underlie also the significance of the ASM-Cer pathway in the pathogenesis of depression. The plasma analyses of patients with major depression over the past 2 years showed an increased level of Cer C16:0, C18:0, C20:0, C24:1, C26:1 compared to those with a past depressive episode more than 2 years ago and versus healthy controls (Gracia-Garcia et al. 2011). Moreover, the cellular activity of the ASM assessed in peripheral blood mononuclear cells is enhanced in depressed patients as compared with healthy subjects. The increased ASM activity may be linked to a rise of inflammatory cytokines (IL-1b, IL-6, and TNF-α) and elevated levels of OS markers (Kornhuber et al. 2005). A recent paper by Rhein et al. (2017) shows that alternative splicing of SMPD1 mRNA, coding for ASM in peripheral blood cells, is present less frequently in depressive patients than in healthy controls. Furthermore, a short 5-day treatment with the FIASMAs fluoxetine or paroxetine, but not with other antidepressant drugs, decreased the frequency of alternatively spliced ASM isoforms in depressive patients. Thus, the ASM alternative splicing pattern could be a biological target with diagnostic relevance and could serve as a novel biomarker for depression (Rhein et al. 2017).

Supporting the above clinical research, many preclinical studies have shown that FIASMA drugs decrease ASM activity (Kölzer et al. 2004; Gulbins et al. 2013; Kornhuber et al. 2011, 2014; Beckmann et al. 2014). Many FIASMAs are clinically effective antidepressant drugs which supports a role of ASM in a major depression. Other preclinical studies conducted in transgenic mice over-expressing ASM (tgASM) showed an increase in the activity of ASM-Cer system seen as higher ASM activity and Cer production in the hippocampus, which was associated with a reduction in neurogenesis and an increase in depression-like behavior (Trapp et al. 2008; Lipinski et al. 2012). The latter actions are mimicked by application of C16-Cer into the dorsal hippocampus () by exposure to various inescapable stressors (Gulbins et al. 2013; Oliveira et al. 2017) or by knockout of CerS1 and CerS6 (Ginkel et al. 2012; Ebel et al. 2013). In line with the view that antidepressants inhibit the activity of ASM, administration of amitriptyline and fluoxetine improved neurogenesis and neuronal survival in the hippocampus and decreased Cer levels in the hippocampus of transgenic mice (Kornhuber et al. 2014).

#### Schizophrenia

Schizophrenia (SZ) is a neuropsychiatric disease, affecting tens of millions people in the world (Young and Geyer 2015). Patients with SZ suffer from various symptoms that fall into three major groups. Positive symptoms include hallucinations and delusions. Negative symptoms include apathy, social withdrawal, and anhedonia. Cognitive symptoms comprise deficits in perception, attention, and working memory. They are closely related to the patient’s ability to function in society (Young and Geyer 2015). The complex etiology of the disease includes genetic, environmental, structural, and functional neurochemical changes in the patient’s brain. The main hypothesis of SZ assumes excessive activity of the DA-ergic system, especially the mesolimbic pathway (Goto and Grace 2007). The co-existence of white matter/myelin diseases suggests that oligodendrocytes and myelin disorders play a key role in the pathogenesis of SZ (Walterfang et al. 2005).

*Post-mortem* analysis of SZ patients has shown that a metabolic dysfunction is particularly notable in the white matter, rather than in the gray matter of the brain. The results indicate the increased level of Cer in the white matter, but not in the gray matter (Prabakaran et al. 2004; Schwarz et al. 2008). In SZ, there is also an impaired metabolism of SM. Patients with SZ have been found to have increased levels of SM in erythrocytes (Keshavan et al. 1993). On the other hand, the level of SM in the thalamus of psychotic patients was considerably reduced (Schmitt et al. 2004). It is worth noting that the altered neuronal content of SM can regulate α7 nicotine acetylcholine receptor activity as a potential target for the treatment of SZ (Colón-Sáez and Yakel 2011).

Increased OS in the acute episode of SZ and disorders of ceramide metabolism may be a by-product of peroxidation of lipid membranes. This proves the genetic abnormality of antioxidant defense in SZ. Scientific studies show a relationship between increased OS and glutamate/DA deficiency in SZ. This may also be due to the involvement of the Cer-SM pathway in regulation of immune function and inflammation (Schwarz et al. 2008).

Future studies should address the question whether current SZ animal models (e.g., PCP rat model) show changes in lipid levels. Up to now, clinical studies suggest some abnormalities in the Cer metabolism. However, whether Cer is a risk factor for SZ has to be confirmed in future studies.

#### Substance Use Disorders

Substance use disorders caused by alcohol consumption, tobacco smoking, and psychoactive drug use are major contributors to the global burden of morbidity and premature death (Gowing et al. 2015). In 2014, 240 million people suffered from alcohol use disorder, 1 billion people smoked tobacco products, 15 million people injected abused substances, while cannabis is the most prevalent illicit drug with 3.5% of the world’s adult population using it (Gowing et al. 2015). Substance use disorder is a brain disorder in which impairments start in the brain reward circuits, including the mesolimbic DA system. Virtually all abused substances enhance glutamatergic transmission in the brain after acute application (Tzschentke and Schmidt 2003). In the in vitro study, it was shown that Cer is a novel modulator of DA transporters and may alter the affinity of DA to DA transporters (Riddle et al. 2003). In line with this observation, clinical and preclinical data support a role of the Cer pathways in Liu et al. (2000) and Reichel et al. (2011). For example, in clinical trials, male alcohol-dependent patients had increased ASM plasma and activity levels as well as Cer d18:1/16:0 and Cer d18:1/18:0 concentrations during detoxification (Reichel et al. 2011). As proposed, alcohol intoxication in humans may be responsible for disturbances in the metabolism of SLs which can serve as biomarkers to improve the diagnosis of patients and to indicate health risks (e.g., depression) associated with alcohol withdrawal (Reichel et al. 2011).

AS demonstrated in the in vitro and in vivo preclinical research, ethanol induces cell death and astroglial damage in cultures and in the developing rat brain, respectively. These effects were associated with activation of ASM and NSM followed by Cer production (Pascual et al. 2003). The Cer-induced apoptotic effects are related to the activation of signaling pathways implicated in cell death (activation of stress-related kinases, c-Jun N-terminal kinase, p38 mitogen-activated protein kinase, and extracellular signal-regulated kinase pathways) (Pascual et al. 2003). Another finding supports the significance of the de novo Cer synthesis with increases in S1P, sphingosine, sphingosine kinase 2, and S1P receptor 1 in ethanol-induced apoptotic neurodegeneration in the 7-day-old mouse brain (cf. Müller et al. 2015).

It was shown that alcohol can be instrumentalized, i.e., used to achieve goals that would be impossible to achieve or require more work load without alcohol use. Alcohol use can serve numerous instrumentalization goals, one of the most important goals being the self-medication for innate or induced psychiatric problems, like for depression and/or anxiety disorders (Müller and Schumann 2011a, b). There is a high comorbidity of depression and alcohol use disorder with bi-directional trajectories. While the neuropharmacology of alcohol is well known, neurobiological mechanisms for alcohol instrumentalization are poorly understood. It was shown that an over-expression of ASM in mice not only induces depression-like behavior (Gulbins et al. 2013), but also enhanced consumption of alcohol and the alcohol-deprivation-effects after repeated withdrawal in a free-choice drinking paradigm. ASM hyperactivity facilitates the establishment of the conditioned behavioral effects of alcohol, and thus, drug-memories. It was shown that free-choice alcohol drinking, but not forced alcohol exposure, reduces depression-like behavior selectively in depressed animals by normalization of ASM activity. ASM hyperactivity induced a sphingolipid- as well as a monoamine transmitter allostasis in the nucleus accumbens. Alcohol drinking restored sphingolipid- and monoamine homeostasis in depressed mice, but had no such effect in wild-type controls. These findings provide the first mechanistic evidence for alcohol instrumentalization with the goal to self-medicate and ameliorate behavioral symptoms of a genetically induced innate depression. From that, sphingolipid homeostasis emerged as a new mechanism to control depression-alcohol addiction comorbidity (Müller et al. 2016).

#### Morphine Antinociceptive Tolerance

Several studies support the view that the ASM/Cer system is an important contributor to the development of morphine analgesic tolerance. Although little is currently known about the mechanism, it was shown that chronic administration of morphine leads to an increase in the production of Cer on the de novo and SM pathways. These effects could be blocked by inhibitors of the Cer biosynthesis, myriocin, and D609 (Bryant et al. 2010). Development of morphine tolerance—with the participation of Cer—results from an activation of opioid μ receptors, neuroimmune activation (TNF-α, IL-1β, and IL-6), and the formation of peroxynitrite (PN). Furthermore, in the periaqueductal gray region of the mice brain, chronic morphine treatment increases the level of ASM and Cer, while the opioid antagonist naloxone or by silencing ASM gene by local plasmid-mediated transfection ASM shRNA blocks the analgesic response to acute morphine as well as the morphine’s action on ASM expression and Cer level. These data indicate that the ASM-Cer pathway in the periaqueductal gray region does play a major role in the antinociceptive mechanism of morphine. In contrast, the impact of ASM shRNA on the development of morphine tolerance is inconclusive (Ritter et al. 2012).

#### Alzheimerʼs Disease

Alzheimerʼs disease (AD) is the most common form of dementia in the elderly. Currently, 24 million people aged 65+ suffer from this disease. It is expected that in 2050, the number of patients will reach 100 million people in the world (Reitz and Mayeux 2014). Some people with gene mutations linked to early-onset AD may experience symptoms at the age of 30+. Early symptoms of AD may include loss of memory, apathy, and depression, while later symptoms include communication disorders, confusion, and behavioral changes like dysphagia (Fargo 2014). AD leads to death within 5 to 9 years after the diagnosis (Niedzielska et al. 2016). The causes of AD are not fully understood. However, it is agreed that the disorder arises from a combination of genetic, lifestyle, and environmental factors that progressively affect the brain. Patients suffering from AD possess less cells and connections among surviving cells as compared to healthy subjects. The regional neuron loss begins in the medial temporal lobe, and later progresses to the hippocampus and cerebral cortex. Two types of abnormalities contribute to AD pathogenesis: tangles and/or plagues. In AD patients, abnormal tangles formed with tau protein are localized inside brain cells. In the hyper-phosphorylated form, tau protein cannot bind to tubulin and microtubule formation leading to a disruption of the neuronal cytoskeleton, a loss of its function, as well as the reduction of microtubuli and neurofilaments. Plagues are composed of amyloid β-protein (Aβ). Dysregulation of Aβ causes insoluble fragments of Aβ proteins containing 40 or 42 amino acid residues inside the neuronal cells and extracellularly in the form of senile plaques. Genes involved in several pathways that are associated with AD pathology include Aβ precursor protein (APP), beta-secretase (BACE1), and presenilin 1 (PS1) (Zuo et al. 2015). Aβ aggregates are toxic and contribute to dysfunction of cell-to-cell communication and neuronal death (Ballard et al. 2011).

In micromolar concentrations, Aβ undergoes a process of nucleation and catalyzes the formation of ROS, such as H_2_O_2_ and hydroxyl radical (^·^OH) (Pietras 2007). The latter process is linked to Aβ-related enhancement of synthesis of—among others—proteins (protein phosphatase 2A (PP2A)), enzymes (e.g., NADPH oxidase), mitochondrial apoptotic proteins (apoptosis inducing factor (AIF), endonuclease G (endo G) and second mitochondria-derived activator of caspases (Smac), and proinflammatory cytokines (by Aβ(25–35)). All these factors enhance the SM–Cer cascade.

Post-mortem analysis of the brain of AD patients showed an abnormal metabolism of Cer (Cutler et al. 2004). Progressively increased levels of Cer in patients with mild to moderate symptoms of AD suggest changes in the metabolism of Cer already at an early stages of the disease and supports SM and Cer as biomarkers of AD (He et al. 2010). Other studies have shown increased levels of Cer, especially Cer 18:1 16:0 and Cer 18:1 24:0, in the plasma of older women being associated with an increase in AD regardless of age, blood glucose, and BMI (Mielke et al. 2012). This suggests that high levels of Cer in the plasma may increase the risk of developing an AD. In the brains of AD patients, a reduced level of S1P can contribute to an increase in ASM activity and in Cer levels (Gómez-Muñoz et al. 2003). Interestingly, under normal circumstances, levels Aβ and Cer maintain a balance in neurons. However, aging and stress can trigger signaling pathways to increase the activity of SM and Cer production. In a vicious circle, increased production of Aβ may occur via Cer that promotes the processing of APP leading to neuronal death (He et al. 2010).

In patients with AD, increased activity of ASM in blood plasma was observed. Similar changes were seen in the brains of mice (He et al. 2010; Lee et al. 2014). Furthermore, in AD transgenic mice, a partial ASM inhibition restores the process of autophagy and leads to a reduction of Aβ deposition as well as to memory deficit improvement. It was proposed that new therapeutic targets should focus on removing Cer, either by inhibiting ASM activity (Young and Geyer 2015) or by increasing the production of S1P (He et al. 2010). Other preclinical studies showed that Aβ(25–35) and Aβ(1–40) activate NSM, but not ASM or Cer synthase, thus leading to the death of oligodendrocytes (OLG) (Lee et al. 2004). The latter effect was prevented by NSM inhibitors showing that a decrease in the Cer synthesis protects OLG against cytotoxic action of Aβ.

#### Parkinson Disease

Parkinsonʼs disease (PD) is a chronic, progressive neurodegenerative disease affecting 1% of the elderly under 60 years of age. Symptoms of the disease appear gradually and develop for several years. Early non-motor symptoms include hyposmia, fatigue, depression, behavioral disorders, and constipation. The primary motor symptoms are bradykinesia, muscle stiffness, rigidity, and resting tremor. Later symptoms include postural instability, dysphagia, anxiety, orthostatic dizziness, urinary incontinence, sweating, and salivation (Connolly and Lang 2014). The etiology of PD is influenced by genetic, environmental, and many other factors. Symptoms of the disease are due to the degeneration of DA-ergic neurons in the brainstem. Lewy bodies are also observed in the areas of the brain affected by pathological changes. These bodies are primarily composed of α-synuclein protein (ASN) and form deposits in the cytoplasm of nerve cells (Sulzer 2007). At the molecular level, observations point to the key role of abnormalities in the functioning and metabolism of ASN (Venda et al. 2010).

Disorders of Cer metabolism can lead to pathogenesis of PD. Limited clinical studies of patients with PD and cognitive impairments showed significantly higher levels of Cer C14:0 and C24:1 in the plasma compared to patients with PD without cognitive impairment or controls. Negative correlations were observed between Cer C14:0 and C24:1 levels and verbal memory. On the other hand, positive correlations were found in PD patients between Cer and hallucinations, anxiety, and sleep disturbances (Xing et al. 2016). Another study has also shown elevated levels of Cer C16:0, C18:0, C22:0, C24:1 in the plasma of patients with PD and cognitive impairments as compared to patients with PD without cognitive impairments or controls (Mielke et al. 2013). Preliminary results suggest that higher levels of Cer in patients with PD are associated with cognitive impairment.

*Post-mortem* analysis of the brain from anterior cingulate cortex (ACC) and occipital cortex (OCC) of PD patients showed an abnormal metabolism of Cer and SM. Studies have shown a significantly increased levels of Cer C18:0 content in the ACC accompanied by a significantly decreased level of Cer C24:1 in PD compared to control. In the OCC, an area spared in PD, this change was not observed. These studies have also demonstrated in the ACC of PD patients decreased levels of SM, e.g., SM C23:0, C24:1, C26:1, compared to controls. In turn, the level of SM C18:1 and C20:0 was increased compared to controls. There were no changes in the levels of SM in the OCC (Abbott et al. 2014). The *post-mortem* results suggest that a dysfunction in Cer and/or SM metabolism is associated with PD. However, further studies on animal models of PD are needed to demonstrate similar changes as in clinical trials and to confirm this hypothesis.

## Conclusion

**Fig. 2:**
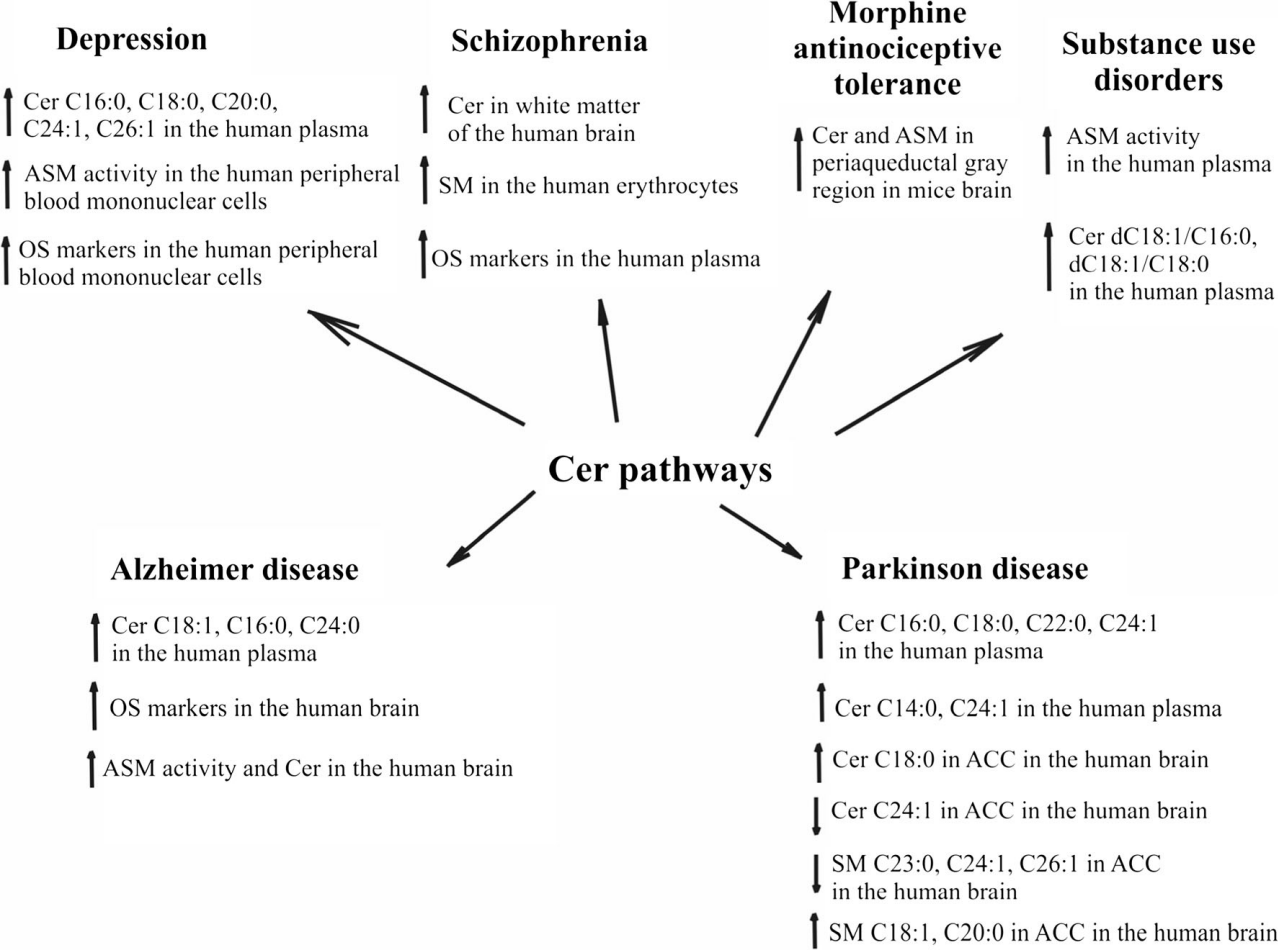
The engagement of OS and Cer pathways in some brain disorders. *ACC* anterior cingulate cortex, *ASM* acid sphingomyelinase, *Cer* ceramide, *OS* oxidative stress, *SM* sphingomyelin

There are currently only limited effective measures for the prevention and treatment of major brain disorders, such as depression, SZ, AD, and PD (See Fig. 2). Thus, there is still a need for new therapeutic strategies and pharmacological approaches. Several studies indicate the role of SLs with a specific acyl chain length in the pathophysiology of nervous system diseases. These results confirm the role of SLs, especially long-chain acyl Cer species in the pathogenesis of major depressive disorders and SZ as well as the neurodegenerative diseases AD and PD. In the case of depression, long-chain acyl Cer species play a role in stimulating the pathogenesis of the disease. However, additional research is needed to determine the mechanisms of Cer metabolism responsible for the pathogenesis of depression. As a result, it may be possible to set new therapeutic goals for the treatment of depression. Disturbance of the metabolism of Cer or SM in neurodegenerative diseases is associated with the pathogenesis of these diseases; however, the exact role of Cer in AD and PD is currently unknown. Current results require further research into Cer metabolism as potential new targets for the prevention or treatment of mental and neurological disorders.
